# Data set on the effects of conifer control and slash burning on soil carbon, total N, organic matter and extractable micro-nutrients

**DOI:** 10.1016/j.dib.2017.08.004

**Published:** 2017-08-05

**Authors:** Jonathan D. Bates, Kirk W. Davies

**Affiliations:** USDA-Agricultural Research Service, Eastern Oregon Agricultural Research Center, 67826-A Hwy 205, Burns, OR 97720, USA

**Keywords:** Western juniper, Calcium, Carbon, Nitrogen

## Abstract

Conifer control in sagebrush steppe of the western United States causes various levels of site disturbance influencing vegetation recovery and resource availability. The data set presented in this article include growing season availability of soil micronutrients and levels of total soil carbon, organic matter, and N spanning a six year period following western juniper (*Juniperus occidentalis* spp. *occidentalis*) reduction by mechanical cutting and prescribed fire of western juniper woodlands in southeast Oregon. These data can be useful to further evaluate the impacts of conifer woodland reduction to soil resources in sagebrush steppe plant communities.

**Specifications Table**

**Table:** 

Subject area	Ecology and Forestry
More specific subject area	Woodland control to restore sagebrush steppe in the Great Basin, USA.
Type of data	Tables, Figures
How data was acquired	Field Collections and laboratory analysis
Data format	Graphically summarized into means and standard errors
Experimental factors	Juniper woodlands were reduced by cutting trees and burning slash in fall winter and spring to determine treatment effects to soil micro-nutrients, total N, C, and organic matter.
Experimental features	Computational analysis of data: percentages, means and standard errors were computed using SAS ver. 9.3 and Microsoft Excel software.
Data source location	Harney County, Oregon, USA; 42° 56′ 10″ N, 118° 36′ 30″ W and 42° 53′ 25″ N, 118° 34′ 18″ W
Data accessibility	Data is with this article and available on request.

**Value of the data**

• This is a unique and long-term dataset of soil nutrient availability, soil carbon (SC), soil organic matter (SOM), and total nitrogen (TSN) after various juniper reduction treatments, which are lacking in the literature.
• The dataset would be useful to researchers comparing short versus longer-term micro-nutrient availabilities following mechanical and prescribed fire disturbance in sagebrush steppe plant communities invaded by conifers.
• The data can be used for multivariate analysis for evaluating nutrient availabilities and vegetation composition (see [2], 2017) at spatial and temporal scales.

## 1 Data

**Fig. 1:**
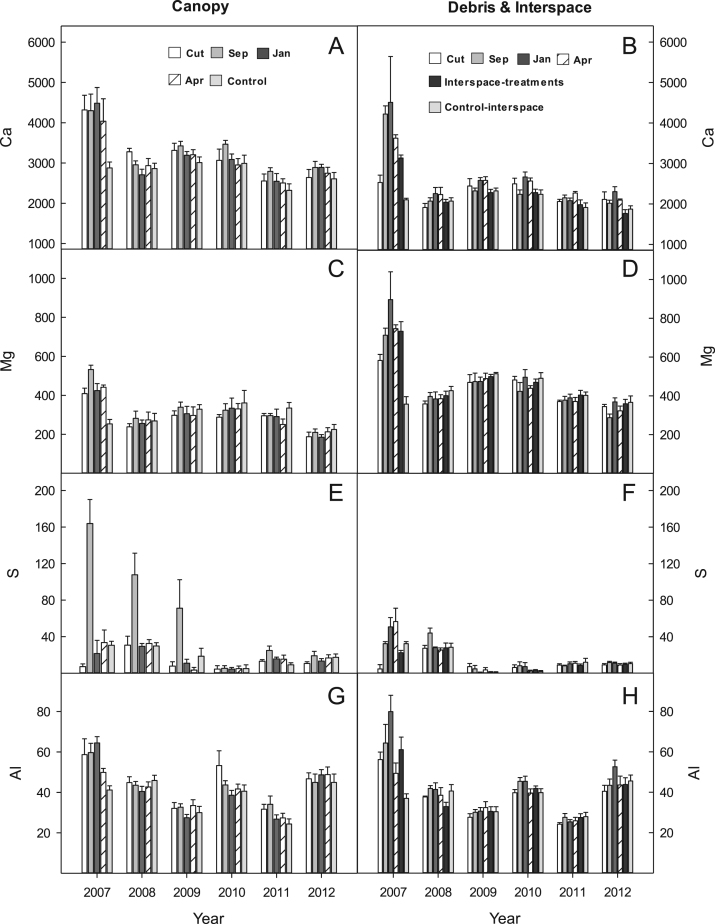
Micronutrient availability (ug 10 cm^2^) for (A & B) Calcium (CA); (C & D) Magnesium (Mg); (E & F) Sulphur (S); and (G & H) Aluminum (Al) for the western juniper control treatments at the BLUEBUNCH site, Steen's Mountain, Oregon, 2007–2012. Data are sorted by canopy and debris-interspace soils and are means + one standard error. Except for CUT and control the other treatment designations correspond to the month burning was applied.

**Fig. 2:**
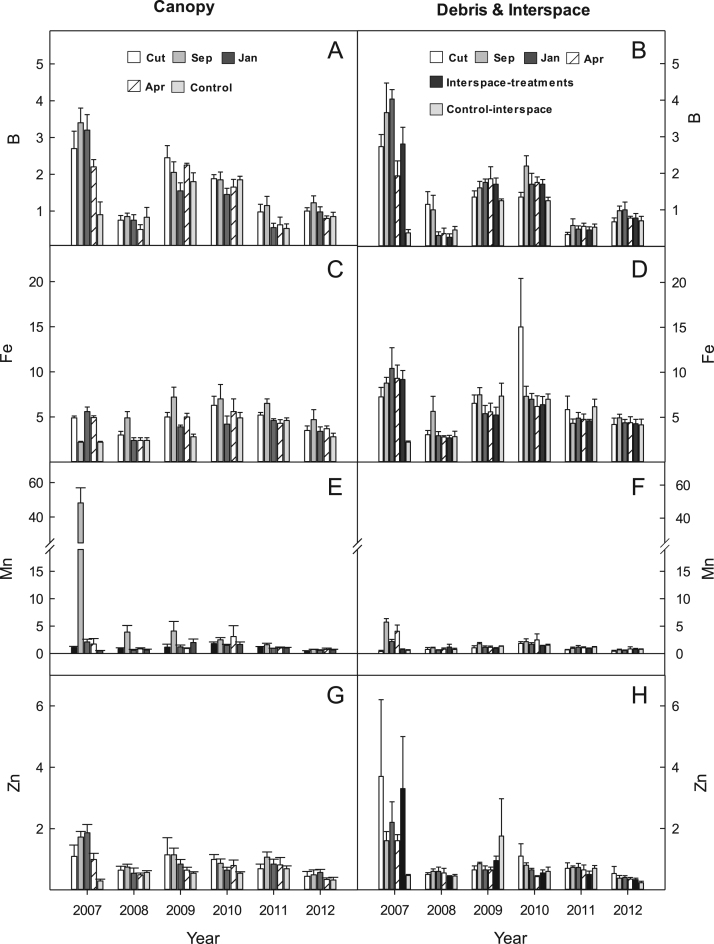
Micronutrient availability (ug 10 cm^2^) for (A & B) Boron (B); (C & D) Iron (FE); (E & F) manganese (Mn); and (G & H0 zinc (Zn) for the western juniper control treatments at the BLUEBUNCH site, Steen's Mountain, Oregon, 2007–2012. Data are sorted by canopy and debris-interspace soils and are means + one standard error. Except for CUT and control the other treatment designations correspond to the month burning was applied.

**Fig. 3:**
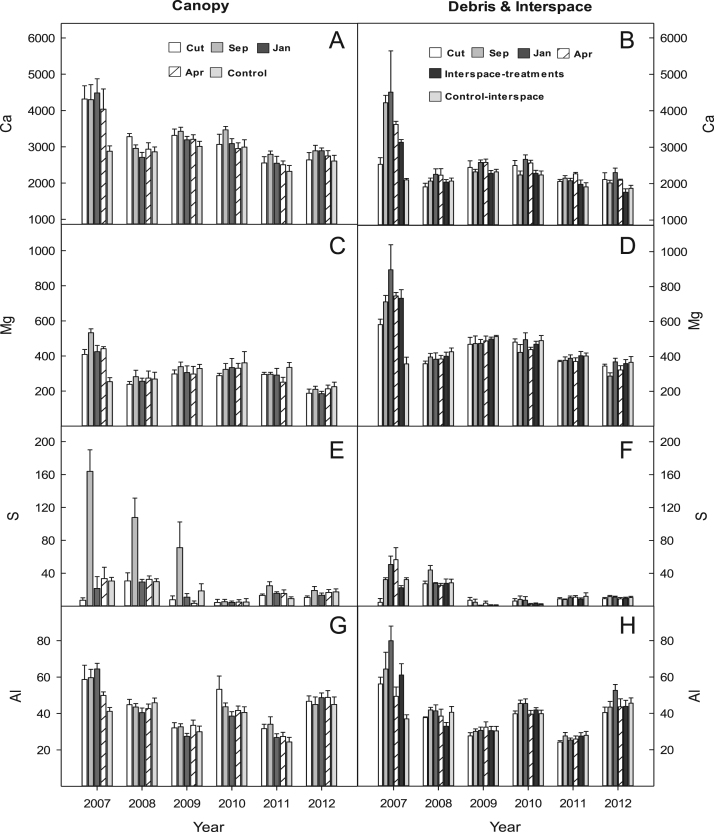
Micronutrient availability (ug 10 cm^2^) for (A & B) Calcium (CA); (C & D) Magnesium (Mg); (E & F) Sulphur (S); and (G & H) Aluminium (Al) for the western juniper control treatments at the FESCUE site, Steen's Mountain, Oregon, 2007–2012. Data are sorted by canopy and debris-interspace soils and are means + one standard error. Except for CUT and control the other treatment designations correspond to the month burning was applied.

**Fig. 4:**
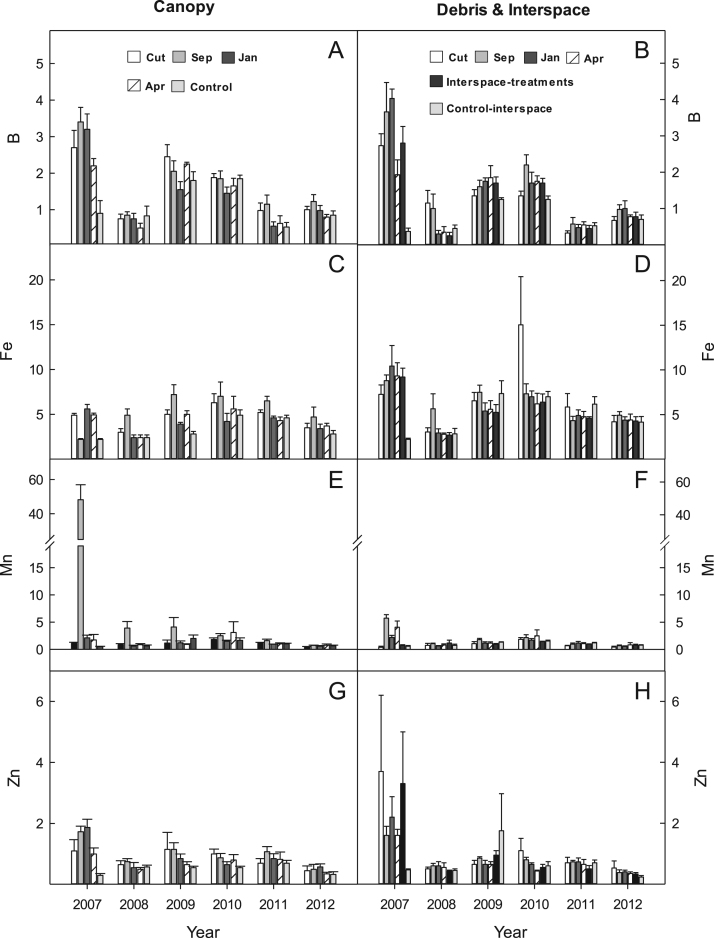
Micronutrient availability (ug 10 cm^2^) for (A & B) Boron (B); (C & D) Iron (FE); (E & F) manganese (Mn); and (G & H) zinc (Zn) for the western juniper control treatments at the FESCUE site, Steen's Mountain, Oregon, 2007–2012. Data are sorted by canopy and debris-interspace soils and are means + one standard error. Except for CUT and control the other treatment designations correspond to the month burning was applied.

**Fig. 5:**
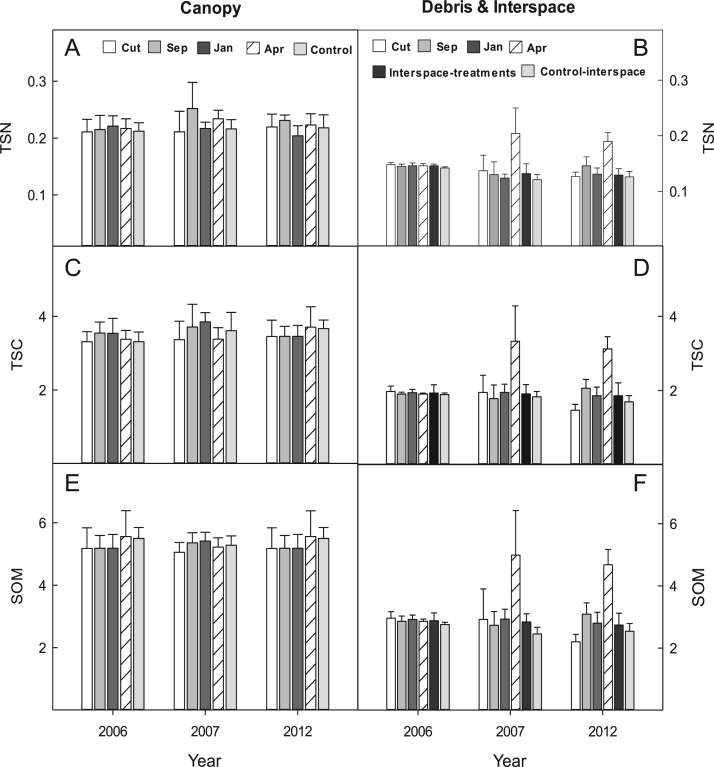
Values (%) for total soil nitrogen (TSN; A & B); total soil carbon (TSC; C & D); and soil organic matter (SOM; E & F) for the western juniper control treatments at the BLUEBUNCH site, Steen's Mountain, Oregon, 2007–2012. Data are sorted by canopy and debris-interspace soils and are means + one standard error. Except for CUT and control the other treatment designations correspond to the month burning was applied.

**Fig. 6:**
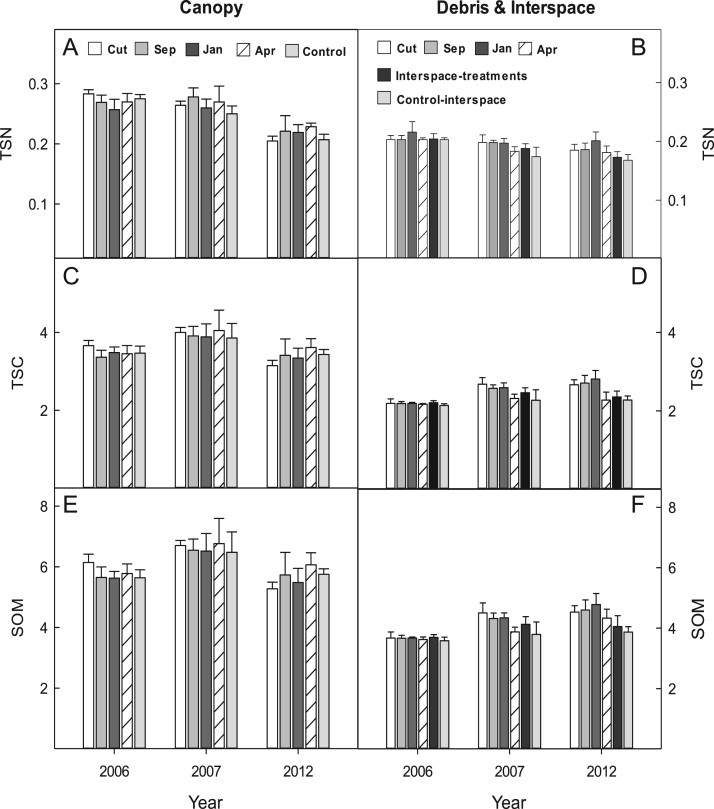
Values (%) for total soil nitrogen (TSN; A & B); total soil carbon (TSC; C & D); and soil organic matter (SOM; E & F) for the western juniper control treatments at the FESCUE site, Steen's Mountain, Oregon, 2007–2012. Data are sorted by canopy and debris-interspace soils and are means + one standard error. Except for CUT and control the other treatment designations correspond to the month burning was applied.

The data shows micronutrient availability, 2007–2012, for two big sagebrush-bunchgrass communities at the BLUEBUNCH site (Fig. 1, Fig. 2), and at the FESCUE site (Fig. 3, Fig. 4) following western juniper control. Soil carbon, SOM, and TSN are presented for the BLUEBUNCH and FESCUE sites in Fig. 5, Fig. 6, respectively. All graphical data are in means and standard errors.

## 2 Experimental design, materials and methods

Two study sites were located on Steen's Mountain, southeast Oregon, 80–90 km south of Burns. The sites were basin big sagebrush/bluebunch wheatgrass-Thurber's needlegrass (*Artemisia tridentata* Nutt. spp. *tridentata* (Rydb.) Beetle/ *Pseudoroegneria spicata* (Pursh) A. Löve - *Achnatherum thurberianum* (Piper) Barkworth) [BLUEBUNCH] and mountain big sagebrush/Idaho fescue (*Festuca idahoensis* Elmer) [FESCUE] plant associations. The BLUEBUNCH and FESCUE sites were classed as Phase 3 woodlands as post-settlement western juniper invasion had eliminated the shrub layer and depleted the understory [1].

The BLUEBUNCH site (42° 56′ 10″ N, 118° 36′ 30″ W) was located on a west aspect (slope 15–22%) at 1550–1600 m. The ecological site is a Droughty Loam 11–13 (280–330 mm) PZ (precipitation zone). Soils are a clayey-skeletal, frigid, Lithic Argixerolls. Soil pH (0–10 cm) was 7.5 in interspace soils and 7.6 under tree canopies. Soil textures (0–10 cm) were a silt-loam in the interspaces and a loam under tree canopies. Bulk density of the soils was 1.2 g cm^-3^ in both the interspace and canopy zones. Prior to treatment, juniper canopy cover averaged 26%, the interspace was 95% bare ground and Sandberg's bluegrass (*Poa secunda* J Pres.), a shallow rooted perennial grass, was the main understory species.

The FESCUE site (42° 53′ 25″ N, 118° 34′ 18″ W) was on an east facing slope (20–45%) at 1650–1730 m. The ecological site was a North Slope 12–16 (304–406 mm) PZ. Prior to treatment, juniper canopy cover averaged 35%, the interspace was 60% bare ground and Idaho fescue and perennial forbs dominated the understory. Soils are a loamy-skeletal, mixed, frigid Pachic Haploxerolls. Soil pH (0–10 cm) was 6.9 in interspaces and 7.2 in tree canopy zones. Soil textures (0–10 cm) were loams and soil bulk density was 1.1 g cm^-3^ in interspace and canopy zones.

The experimental design at each site was a randomized complete block with three cut-and-burn treatments, a cut-and-leave (CUT) treatment, and woodland controls. There were five treatment replicates at each site. Treatment plots ranged from 0.2 to 0.4 ha in size. Cut-and-burn treatments included fires applied in September (SEP), January (JAN), and April (APR). All juniper in the JAN, APR, and CUT treatments were felled in July, 2006. JAN fires were applied on 17 and 19 Jan, 2007, on the BLUEBUNCH, and FESCUE sites, respectively. These fires were rated at low severity [1], [2], [3]. APR fires were applied on 6 Apr 2007 at both sites and fires were of low (interspace) to high (beneath cut trees) severity. JAN and APR burns required igniting individual or clusters of trees as snow or green herbaceous vegetation prevented fire from carrying in the interspaces. On SEP treatments one-third of the juniper were cut in June 2006 and once dry were used to carry strip-head fires to kill remaining live trees. The SEP fires were of moderate to high severity and were applied on 25 and 26 Sep, 2006, at the BLUEBUNCH and FESCUE sites, respectively. Burn conditions were typical for applications used to broadcast burn (SEP) and reduce western juniper fuel loads in winter and spring [1].

Flame lengths, burn duration, area burned, soil temperatures and fuel consumption were lowest in JAN treatments and greatest in SEP treatments [1]. Juniper fuel consumption in canopy and felled tree zones of SEP and APR treatments consumed all 1-h, 10-h and 100-h fuels and partly consumed the 1000-h fuels. In interspaces of SEP treatments herbaceous fine fuels were consumed as were scattered shrubs. Interspace zones in APR treatments did not burn though heat damage was noted for plants in close proximity to burning trees. In JAN treatments, fire only consumed 1-h fuels in the felled tree zones while canopy and interspace zones did not burn.

Concentrations of soil micronutrients, were collected using Plant Root Simulator probes (PRS™-probes; Western Ag Innovations, Saskatoon, Saskatchewan, Canada) for each treatment in three blocks at each site. Ion-exchange resin membranes on the PRS™-probes collect anions and cation in the soil solution using electrostatic attraction. The PRS™-probes were planted vertically below the soil surface with the membrane section of the probe collecting ions from 1.3 to 6.8 cm deep. Probes were left in situ from early to mid-April until late July, each year, from 2007 to 2012. Probes were not isolated and plant roots could compete with the ion resins for soil nutrients. Micronutrients include Aluminum (Al), Boron (B), Calcium (Ca), Iron (Fe), Magnesium (Mg), Manganese (Mn), Sulphur (S), and Zinc (Zn). At the end of the sample period, probes were collected, washed with deionized water, locked in labeled polyethylene bags, and returned to Western Ag Innovations where they were extracted with 0.5 N HCl and analyzed either colourimetrically with an auto-analyzer or plasma emission spectroscopy to determine nutrient concentrations. Nutrient availability was measured in three zones; interspace, litter mats beneath formerly standing trees (canopy), and beneath felled trees (debris). The debris zone was former interspace overlain by felled trees. Four probe sets (one set is one cation and one anion probe) were randomly placed in each zone within a treatment plot. On control plots probes were placed in canopy (beneath live trees) and interspace zones.

Total soil nitrogen (N), carbon (SC), and organic matter (SOM) in the upper 10 cm of the soil profile were determined from five soil samples, collected in July 2006, 2007, and 2012 from each treatment zone per block. SC and N were determined using a LECO CN 2000. SOM was estimated using an amended Rather method.
