# Supplementary material for: Data set on the effects of conifer control and slash burning on soil carbon, total N, organic matter and extractable micro-nutrients
Source: Data Brief. 2017 Aug 5;14:515–23. doi: 10.1016/j.dib.2017.08.004 (PMC5562109; doi:10.1016/j.dib.2017.08.004)
Supplement: Supplementary file 1 — Supplementary material [file mmc1.pdf]

Conflicts of Interest Statement

Manuscript title: Dataset on the effects of conifer control and slash burning on soil carbon, total N, organic matter and extractable micro-nutrients

The authors whose names are listed immediately below certify that they have NO affiliations with or involvement in any organization or entity with any financial interest (such as honoraria; educational grants; participation in speakers' bureaus; membership, employment, consultancies, stock ownership, or other equity interest; and expert testimony or patent-licensing arrangements), or non-financial interest (such as personal or professional relationships, affiliations, knowledge or beliefs) in the subject matter or materials discussed in this manuscript.

Author names:

Jonathan D. Bates

Kirk W. Davies

NO CONFLICTS

The authors whose names are listed immediately below report the following details of affiliation or involvement in an organization or entity with a financial or non-financial interest in the subject matter or materials discussed in this manuscript. Please specify the nature of the conflict on a separate sheet of paper if the space below is inadequate.

Author names:

This statement is signed by all the authors to indicate agreement that the above information is true and correct (*a photocopy of this form may be used if there are more than 10 authors*):

Author's name (typed)	Author's signature	Date
Jonathan D. Bates	Jonathan D. Bates	7-14-2017
Kirk W. Davies	[illegible]	7-14-17
